# Oropharyngeal dysphagia management in cervical spinal cord injury patients: an exploratory survey of variations to care across specialised and non-specialised units

**DOI:** 10.1038/s41394-019-0175-y

**Published:** 2019-04-15

**Authors:** Jackie McRae, Christina Smith, Suzanne Beeke, Anton Emmanuel

**Affiliations:** 1 Division of Medicine, University College London, WC1E 6JF London, UK; 2 Speech and Language Therapy Service, London Spinal Cord Injury Centre, Royal National Orthopaedic Hospital, Stanmore, HA7 4LP London, UK; 3 Division of Psychology and Language Science, University College London, WC1N 1PF London, UK

**Keywords:** Rehabilitation, Trauma

## Abstract

**Study design:**

A multi-centre online survey to staff working in specialised and non-specialised acute units.

**Objectives:**

To identify clinical decisions and practices made for acute cervical spinal cord injury (CSCI) patients with respiratory impairments and oropharyngeal dysphagia.

**Settings:**

All hospital intensive care units in the UK that admit acute cervical spinal cord injury patients.

**Methods:**

Online distribution of a 35-question multiple-choice survey on the clinical management of ventilation, swallowing, nutrition, oral hygiene and communication for CSCI patients, to multi-disciplinary staff based in specialised and non-specialised intensive care units across UK.

**Results:**

Responses were received from 219 staff members based in 92 hospitals. Of the 77 units that admitted CSCI patients, 152 participants worked in non-specialised and 30 in specialised units. Non-specialised unit staff showed variations in clinical decisions for respiratory management compared to specialised units with limited use of vital capacity measures and graduated weaning programme, reliance on coughing to indicate aspiration, inconsistent manipulation of tracheostomy cuffs for speech and swallowing and limited use of instrumental assessments of swallowing. Those in specialised units employed a multi-discplinary approach to clinical management of nutritional needs.

**Conclusions:**

Variation in the clinical management of respiratory impairments and oropharyngeal dysphagia between specialised and non-specialised units have implications for patient outcomes and increase the risk of respiratory complications that impact mortality. The future development of clinical guidance is required to ensure best practice and consistent care across all units.

## Introduction

Demographics of SCI have changed in western countries from young men involved in high-velocity crashes towards older people with low-velocity falls [1]. This has led to increased rates of SCI at the cervical level [2] with associated paralysis of respiratory muscles and need for ventilatory support via tracheostomy. The disruption to respiratory mechanics and laryngeal function is linked to the disruption of normal swallowing function and oropharyngeal dysphagia, with a reported incidence of 30–40% increasing morbidity and mortality rates [3, 4].

In the UK, national guidance for ventilator weaning of CSCI patients was developed through consensus by a multi-professional group called Respiratory Information for Spinal Cord Injury (RISCI) [5]. This recommends a graduated process of respiratory weaning, using vital capacity (VC) as a key measure of respiratory fatigue. Periods of ventilator-free breathing will be determined based on VC with prescribed rest periods to limit fatigue due to retraining. Early gastrostomy is recommended for those who are likely to wean slowly and subglottic tracheostomy tubes support secretion clearance. Deflation of the tracheostomy cuff is encouraged to facilitate swallowing [6, 7] and speaking using a one-way valve, especially in the critical care environment. It is acknowledged that the weaning process can take an extended time with potential setbacks that demand a collaborative team approach. This guidance has been agreed and adopted by teams in specialised spinal units however it is not known whether staff in non-specialised units adhere to these recommendations. There is no specific guidance available on the clinical management of oropharyngeal dysphagia following CSCI.

The optimal management of respiratory, nutrition and swallowing problems requires involvement from multiple clinical professionals, including doctors, nurses, physiotherapists, speech and language therapists and dietitians. The aim of this study was to explore the clinical practices of multi-disciplinary staff within specialised and non-specialised critical care units in the management of complex CSCI patients with respiratory and swallowing disorders. This information would highlight variations in care and help to contribute to future development of best practice recommendations to ensure consistent clinical management. This is the first study in a series of studies contributing to a doctoral investigation into the identification and management of oropharyngeal dysphagia in acute cervical spinal cord injury (DAISY project).

## Methods

Owing to the absence of any pre-existing multi-disciplinary survey on the management of CSCI patients, a new survey was developed through a process of literature review to identify topic areas followed by survey construction and piloting with a representative group for validation.

### Survey design and development

Topics for inclusion were derived from a review of the literature on oropharyngeal dysphagia, respiratory function, nutrition, oral care and communication in CSCI. A Medline search was performed using the terms “dysphagia”, “deglutition”, “ventilator weaning”, “tracheostomy”, “respiratory”, “enteral nutrition”, “oral hygiene” and “communication” each of which were paired with “spinal cord injury”, “cervical” and “tetraplegia”. Searches were limited to studies with human adults, written in English.

In devising the survey, multiple-choice questions were devised across five topic areas, with an option for free text comments. The topics were ventilator and tracheostomy weaning, nutritional decisions, dysphagia management, mouthcare and communication support.

A total of 35 questions were created across 22 pages with two additional free-text questions (see supplementary material). Adaptive questioning was used for two questions: grading differences between doctors, nurses and allied health professionals (AHPs) and mouthcare involvement. All professional groups were asked the same survey questions. The content and structure of the survey was evaluated by a steering group of representative multi-disciplinary professionals with expertise in acute SCI and amendments were made subject to their feedback.

### Sample selection

Multi-professional respondents were sought from all critical care units in UK that admitted spinal cord injury patients, this included major trauma centres (MTC), district general hospitals (DGH), teaching hospitals (TCH), spinal injury units (SIU) and specialist hospitals (SPH) such as neurological or cardiothoracic units. Although it was expected that SCI patients would be admitted directly to one of 22 MTC, referrals to SIUs came from a broad range of hospitals, however it was not possible to identify all these units in advance. For this reason, purposive sampling was employed through email distribution to members of each critical care network and relevant professional bodies in UK. This included the Intensive Care Society, British Association of Critical Care Nurses (BACCN), Royal College of Speech and Language Therapists (RCSLT), British Dietetic Association (BDA) and The Chartered Society of Physiotherapy (CSP) critical care groups. To increase recruitment of multi-professionals within and across units, snowball sampling was employed, whereby study participants were asked to invite other colleagues to respond to the survey.

Limited personal demographic details were collected, including profession, grade and hospital name, which was used to categorise unit type. Hospitals described as MTC, DGH and TCH were sub-categorised as non-specialised units as they did not specialise in SCI care, whereas SPH and SIU were considered specialised units, where the focus was only on SCI or neurological care.

### Survey administration and analysis

The survey was launched in August 2014 and closed in January 2015. Responses were collected through an online web survey with results analysed using SPSS Statistics version 22 (IBM) to generate descriptive data. This included the total number and percentage of responses per question and responses per hospital type. To explore variations in the care delivered to CSCI patients, responses were grouped into those from specialised and non-specialised hospitals. Responses from staff working in a SIU or SPH were grouped as specialised hospitals, while those responses from MTC, DGH and TCH were grouped as non-specialised hospitals (Table 1).

**Table 1:** Survey respondents per hospital type

Hospital type	Total respondents *n* = 219	%	Respondents in units admitting CSCI *n* = 182	%	Non-specialised unit respondents	Specialised unit respondents
MTC	88	40	83	46	152	–
DGH	59	27	43	24		
TCH	31	14	26	14		
SIU	29	13	26	14	–	30
SPH	12	6	4	2		

*CSCI* cervical spinal cord injury, *MTC* major trauma centre, *DGH* district general hospital, *TCH* teaching hospital, *SIU* spinal injury unit, *SPH* specialist hospital

## Results

### Respondent demographics

**Fig. 1:**
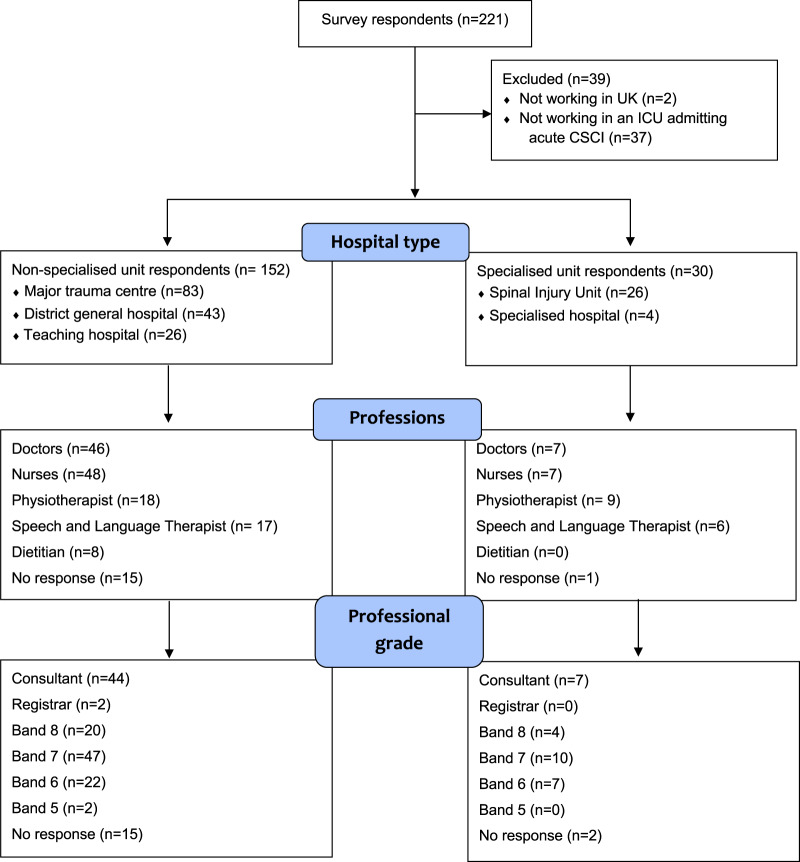
Flow Diagram of survey respondent demographics

A total of 221 respondents participated in the survey (Fig. 1). Two overseas responses were excluded leaving a total of 219 multi-professional respondents from 92 units in UK. Respondent numbers varied across each hospital type and survey analysis was limited to 182 respondents from 77 units that admitted acute spinal cord injury patients, including eight spinal units in England, one from Scotland and one from Ireland, the remainder were excluded from survey completion. All five professional groups were represented in the responses from non-specialised units whereas specialised units lacked dietetic respondents. Medical respondents were senior and experienced while non-medical staff respondents represented a range of grades.

### Clinical practices

These results focus on the clinical practices in the management of respiratory impairment, dysphagia, nutrition and communication for CSCI patients as reported by staff in non-specialised and specialised units. Owing to variations in group sizes, comparisons between units will be based on the typical practice reported.

Respondents from non-specialised units reported links to at least one spinal outreach team in England to access specialist advice and support. Eight respondents had links to spinal outreach teams in Glasgow or Dublin. Some respondents accessed more than one unit and five reported no known links to a spinal outreach service (Table 2).

**Table 2:** Access to spinal outreach team at specified spinal injury unit

	Non-specialised unit responses *n* = 152^a^	%
London Spinal Cord Injury Centre, Royal National Orthopaedic Hospital, Stanmore	37	27
National Spinal Cord Injury Centre, Stoke Mandeville Hospital, Aylesbury	33	24.1
Princess Royal Spinal Injuries Centre, Northern General Hospital, Sheffield	26	19
The Golden Jubilee North East Regional Spinal Injuries Centre, James Cook Hospital, Middlesbrough	20	14.6
Midlands Centre for Spinal Injuries, Robert Jones & Agnes Hunt Hospital, Oswestry	14	10.2
North West Regional Spinal Injuries Centre, Southport & Formby Hospital	11	8
Duke of Cornwall Spinal Treatment Centre		
Salisbury District Hospital, Salisbury	9	6.6
The Yorkshire Regional Spinal Injuries Centre, Pinderfields Hospital, Wakefield	4	2.9
None	5	3.6
Other	8	5.8

^a^Includes multiple responses

### Respiratory management of CSCI patients

Staff in specialised units utilised a number of methods to facilitate ventilator and tracheostomy weaning (Table 3). Vital capacity measures were routinely used to guide the respiratory weaning process, alongside cuff deflation, speaking valves and subglottic suction tubes, which were used less frequently by staff in non-specialised units. Routine tracheostomy capping prior to decannulation was strongly preferred by staff in specialised units. This forms the basis of the RISCI guidelines however few non-specialised unit staff used this guidance or outreach advice and some were uncertain about whether they used any guidance (Table 3).

**Table 3:** Respiratory management of CSCI patients

	Non-specialised units *N* = 152	%	Specialised units *N* = 30	%
*Ventilator weaning process*				
Cuff deflation^a^	103	67.8	25	83.3
Speaking valve^a^	96	63.2	23	76.7
Trache mask	85	55.9	15	50
Vital capacity^a^	51	33.6	23	76.7
Fenestrated tube	46	30.3	9	30
Suctionaid tube^a^	43	28.3	10	33.3
Don’t know	8	5.3	0	0
Other	6	3.9	4	13.3
*Weaning protocol*				
Locally agreed protocol	52	34.2	12	40
Spinal outreach team protocol	23	15.1	7	23.3
National guidance^a^	7	4.6	4	13.3
Don’t know	25	16.4	2	6.7
None	15	9.9	1	3.3
Other	3	2	1	3.3

^a^Recommendation of RISCI guidance

### Oropharyngeal dysphagia identification and management

Staff in specialised and non-specialised units reported the key signs of the presence of oropharyngeal dysphagia in CSCI patients to be coughing, food suctioned from the tracheostomy with clinical symptoms being aspiration pneumonia, spiking pyrexia and wet sounding voice (Table 4). Methods for screening for oropharyngeal dysphagia involved monitoring saliva clearance, swallow trials with water and yoghurt. Staff also reported using blue dye and thickened fluids to test of swallowing. Routine swallow assessment used by Speech and Language Therapists was reported to be bedside swallow evaluations. The use of instrumental swallowing assessments, namely Fibreoptic Evaluation of Swallowing (FEES) and Videofluoroscopy (VFS), were reported as used more frequently by staff in specialised units (Table 4).

**Table 4:** Management of oropharyngeal dysphagia

	Non-specialised units *N* = 152	%	Specialised units *N* = 30	%
*Identification of dysphagia*				
Coughing or choking	110	72.4	25	83.3
Food suctioned from tracheostomy	104	68.4	26	86.7
Aspiration pneumonia	102	67.1	26	86.7
Patient complaint of dysphagia	83	54.6	21	70
Wet voice	69	45.4	21	70
Intra-oral food residue	70	46.1	18	60
Dropping O2 saturations	68	44.7	19	63.3
Spiking pyrexia	35	23	14	46.7
Patient complaint of throat pain	18	11.8	8	26.7
Dysphagia not expected	3	2	1	3.3
Other	6	3.9	0	0
*Swallow screening*				
Saliva	71	46.7	17	56.7
Water	73	48	14	46.7
Thickened fluids	52	34.2	8	26.7
Blue dye	51	33.6	8	26.7
Yoghurt	44	28.9	10	33.3
Speaking	27	17.8	5	16.7
Other	17	11.2	5	16.7
Don’t know	4	2.6	3	10
*Instrumental assessment*				
BSE	99	65.1	23	76.7
FEES	37	24.3	14	46.7
VFS	29	19.1	12	40
ENT Flexible nasendoscopy	9	5.9	2	6.7
Don’t know	9	5.9	0	0
*Eat and drink with cuff inflated*				
Sometimes	64	42.1	11	36.7
No^a^	22	14.5	12	40
Yes	18	11.8	0	0
Don’t know	5	3.3	2	6.7
Other	7	4.6	1	3.3

^a^Recommendation of RISCI guidance

Speech and Language Therapists (SLT) are the staff members to manage oropharyngeal dysphagia in UK and approximately half of staff reported routine availability of SLT services. Referral to SLT was usually made after a positive nurse swallow screen test although aspiration pneumonia was a more frequent cause for referral by staff in specialised units. With some debate about patients eating with tracheostomy cuff inflated, this was reported as happening either routinely or sometimes by staff in non-specialised units whereas staff in specialised unit only allowed cuff up eating occasionally (Table 4).

### Nutritional management

Staff at specialised and non-specialised units agreed on similar criteria for determining the need for non-oral feeding (Table 5). Having a tracheostomy in situ was reported more by staff in non-specialised units. The decision to transition to long term gastrostomy feeding tubes differed between units, staff in specialised units adhered to recommendations by SLT and dietitians, whereas staff in non-specialised units based their decisions on whether swallowing problems were ongoing (Table 5). Staff relied on repeat swallow assessments to determine whether a patient was safe to return to oral intake.

**Table 5:** Nutritional management

	Non-specialised units *N* = 152	%	Specialised units *N* = 30	%
*Reason for non-oral feeding*				
Unable to meet nutritional requirements orally	96	63.2	23	76.7
Prolonged intubation	61	40.1	12	40
Prolonged sedation	57	37.5	12	40
Tracheostomy in situ	33	21.7	1	3.3
Can’t sit upright	15	9.9	4	13.3
Infection	8	5.3	2	6.7
Don’t know	6	3.9	1	3.3
Other	7	4.6	3	10
*Reason for transition from NGT to PEG*				
Ongoing swallowing problems	87	57.2	21	70
SLT recommendation	69	45.4	22	73.3
NG in-situ 4–6 weeks	58	38.2	16	53.3
Dietitian recommendation	52	34.2	21	70
Patient discomfort	35	23	10	33.3
Repeated displacement	37	24.3	8	26.7
Assist hospital transfer	23	15.1	3	10
Increased nutritional need	7	4.6	1	3.3
Infection risk	4	2.6	2	6.7
Don’t know	9	5.9	1	3.3
Other	6	3.9	3	10

*NGT* nasogastric tube, *PEG* percutaneous endoscopic gastrostomy

### Communication

Overall, staff at both specialised and non-specialised units tended to use low-technology methods to facilitate patients’ ability to communicate, with advice given to patients and families and encouragement to use mouthing to help express themselves. High-technology aids were not used as frequently, as physical access is limited. Using cuff deflation to allow leak speech was more consistently employed by specialised unit staff along with in-line speaking valves (Table 6).

**Table 6:** Communication options

	Non-specialised units *N* = 152	%	Specialised units *N* = 30	%
*Communication options*				
Low-technology aids	99	65.1	21	70
Advice to patients and family	97	63.8	22	73.3
Encourage mouthing	88	57.9	22	73.3
High-technology aids	48	31.6	9	30
No special support	2	1.3	0	0
Don’t know	5	3.3	1	3.3
Other	6	3.9	2	6.7
*Cuff down for speech*				
Yes^a^	51	33.6	18	60
Sometimes	44	28.9	4	13.3
No	10	6.6	3	10
Don’t know	10	6.6	1	3.3
*Use of speaking valves*				
Sometimes	66	43.4	14	46.7
No	27	17.8	3	10
Always^a^	15	9.9	6	20
After nasendoscopy	0	0	1	3.3
Other	8	5.3	1	3.3

^a^Recommendation of RISCI guidance

## Discussion

This is the first exploratory study to investigate the clinical care of respiratory dysfunction, oropharyngeal dysphagia and nutrition in CSCI patients within specialised and non-specialised units across the UK. Systematic reviews of the care provision in SIUs support early admissions both to prevent complications and improve outcomes [8, 9]. Deteriorating respiratory function is frequently cited as a key complication with pneumonia contributing to mortality [10, 11]. Oropharyngeal dysphagia increases the risk of aspiration pneumonia, which is likely to add burden to existing respiratory dysfunction [12, 13]. The need for this study was based on the increasing delays to admission to specialised units, particularly for those with cervical level injuries and associated respiratory requirements. This is contrary to the nationally set algorithm for acute SCI care, suggesting prompt transfer to a specialised unit following an MTC admission in order to access the required specialist interventions [14]. A recent report has identified increasing demand and limited bed capacity in specialised units resulting in CSCI patients remaining in non-specialised units for prolonged acute care [15]. In the absence of clinical guidance, this survey aimed to identify clinical practices that have an impact on patient outcomes and would benefit from clear clinical guidance.

The evidence for the pathophysiology of oropharyngeal dysphagia following CSCI is unclear as studies are largely observational and retrospective. Respiratory, neurological and mechanical disruption to the cervical region appear to contribute to laryngeal dysfunction. Early case series reported links to dysphagia although the causes were unclear. Pollock et al. [16] reported four cases of unexpected pharyngeal damage post cervical trauma, while Grundy et al. [17] highlighted the presence of ‘bulbar palsy, with acute respiratory distress and dysphagia’’ in eight patients with cervical injuries. In a larger review, Hsu et al. [18] identified 47 cases of glottis or tracheal stenosis over a 20-year period, causing dysphagia, dysphonia and excessive secretions. Although the cranial nerves in the brainstem innervate many facial and laryngeal functions, there is evidence for anastomoses with the cervical spinal nerves creating a cervical plexus [19]. The ansa cervicalis provides motor innervation to the muscles control hyoid function, essential for swallowing and speech [20]. Fibres of the hypoglossal nerve also join the ansa cervicalis to innervate part of the tongue. The variability in the location of these structures may partially explain the loss of swallow function following CSCI [21].

The loss of phrenic nerve function with injuries above C5, interrupts normal breathing patterns and paralyses the diaphragm. Swallowing function is closely coordinated with the breathing cycle and disruption leads to accidental inhalation and aspiration during the swallow, with lack of cough preventing airway clearance [22]. Respiratory interventions including tracheostomy insertion and supported ventilation are known to cause additional disruption to swallowing and are frequently cited as factors linked to dysphagia following CSCI [3, 4, 23–26].

Cervical spinal surgery is often indicated for traumatic injuries and much of the evidence from elective surgery cohorts report both dysphagia and dysphonia as common post-operative complications often due to pharyngeal wall oedema and nerve injury due to retraction time [27–29], with both posterior and anterior approaches demonstrating neurological impact on swallowing function [30].

Gastrointestinal functions are often affected following CSCI due to the loss of autonomic control, leading to dysmotility and paralytic ileus, often requiring aspiration of gastric contents [31]. This adds another level of challenge for patients identified with dysphagia as alternative routes of nutrition need to be considered. High malnutrition rates have been reported in SCI populations admitted to specialised units in England [32] of which CSCI and tracheostomy patients were the largest cohort. Decisions about early nutritional support appear to be inconsistent and rely on a wait-and-see approach and evidence of prolonged oropharyngeal dysphagia rather than pro-active management.

The combination of respiratory, swallowing and gastrointestinal impairment increases the risk of aspiration and staff need to be aware of the signs in order to prevent symptoms developing, which can be difficult to reverse. The survey results demonstrate variations in care that can have an impact on these functions and the negative consequences of dysphagia. A graduated weaning approach, utilising vital capacity as a clinical measure, has been recommended for CSCI patients through expert consensus and adopted by the Intensive Care Society [5], however, this is not routinely used by staff in non-specialised units.

Accurate evaluation of laryngeal dysfunction helps the weaning process and permits more options for verbal communication, which is important for intensive care patients [33]. Currently there is little guidance to direct staff on optimal management of tracheostomy [34] or dysphagia and some methods described by staff may put patients at increased risk of aspiration pneumonia, dehydration and mortality [35, 36]. This includes the use of thickened fluids for patients with dysphagia, on the premise that increased viscosity slows transit of the fluid allowing more time for a delayed swallow initiation to capture the bolus. In contrast, the key feature of dysphagia in CSCI patients is not delayed swallowing but ineffective pharyngeal squeeze [26] making thickened fluids unsuitable as they are more challenging to clear from the pharynx and can increase damage to lung mucosa due to aspiration [37]. Similarly, a reliance on the cough reflex to signify aspiration is problematic especially for patients with inflated tracheostomy cuffs [38] or absent cough ability due to impairment to the vagus nerve [39]. Instead, instrumental assessments, such as FEES or VFS are recommended to identify laryngeal impairments that are asymptomatic at bedside [3, 23, 24, 26, 40].

As with many surveys, there were limitations to using this method of evaluating current practice. Recruitment of multi-professional staff in specialised and non-specialised units relied on purposive and snowball sampling, making a response rate difficult to calculate. However, responses from staff at a large number of units across UK did reveal the widespread transfer of SCI patients from trauma units to non-specialised units, which does not fulfil the national service specification for acute SCI management and this would benefit from further investigation and analysis of the national database. Sample sizes for each group varied considerably making statistical comparisons inappropriate, instead the results provide qualitative data and insight into current practices, which have not previously been identified. Some professional groups were less well represented and staff reported that they had little disease-specific training and expertise with this patient group, despite having to deliver care. This may have had an impact on the survey completion rate with a loss of just over a third (35%) of respondents. A follow-up study may consider profession specific questionnaires or interviews, to explore practices in more detail.

The incidence of CSCI are increasing nationally [1, 41] and are often associated with respiratory dysfunction and oropharyngeal dysphagia [42]. With limited bed availability in specialised units in UK, these patients remain in non-specialised units for management of these impairments. This survey highlighted variations in clinical practice in the absence of national guidance, which have an impact on clinical outcomes. With no plans to increase respiratory bed capacity in specialised units, clinical guidance is required to support multi-disciplinary teams to deliver safe and consistent care. Further research is needed to capture the clinical outcomes for CSCI patients managed in specialised and non-specialised units.

## Disclaimer

This paper presents independent research funded by the National Institute for Health Research (NIHR) and Health Education England. The views expressed are those of the authors and not necessarily those of the NHS, the NIHR or the Department of Health and Social Care.

## Supplementary information

Respiratory Information for Spinal Cord Injury UK (RISCI) weaning guidelines for spinal cord injured patients in critical care units

Online survey questions
