# Supplementary material for: Oropharyngeal dysphagia management in cervical spinal cord injury patients: an exploratory survey of variations to care across specialised and non-specialised units
Source: Spinal Cord Ser Cases. 2019 Apr 15;5:31. doi: 10.1038/s41394-019-0175-y (PMC6474233; doi:10.1038/s41394-019-0175-y)
Supplement: Supplementary file 1 — Respiratory Information for Spinal Cord Injury UK (RISCI) weaning guidelines for spinal cord injured patients in critical care units [file 41394_2019_175_MOESM1_ESM.docx]

| **Supplementary material: Survey questions** |
| --- |
| Section 1 Hospital and unit details: |
| 1. What type of hospital do you work in? |
| 2. How many beds does the hospital have? |
| 3. What is the main hospital ICU that you work in? |
| 4. Does your intensive care unit have established links with a .. |
| 5.How many level 3 beds does your intensive care unit have? |
| 6.Does your intensive care unit admit patients with spinal cord injury? |
| 7. Does your unit have specific care pathways for managing: |
| 8.Which Spinal Outreach Team does your unit have access to? |
| Section 2: Demographic details |
| 9. What profession are you? |
| 10. What grade are you? OR 11. What banding are you? |
| 12. What is your clinical specialism? |
| Section 3: Ventilator and tracheostomy weaning |
| 13. Who is regularly involved in your tracheostomy team? |
| 14. Who determines the weaning programme of ventilated spinal cord injured patients? |
| 15. What protocol does the team use for ventilator weaning of patients with cervical spinal cord injury? |
| 16. What is the first priority when planning a ventilator weaning programme? |
| 17. As part of the ventilator weaning process do you use: |
| 18. What do you consider is the impact of an inflated tracheostomy cuff? |
| 19. Do you routinely block off a tracheostomy before decannulation? |
| Section 4: Feeding |
| 20. What criteria does your unit use for commencing non­oral feeding in a spinal cord injured patient? |
| 21. What will determine the change from a nasogastric tube to a gastrostomy feeding tube? |
| 22. When do you consider a cervical spinal cord injured patient is ready to start eating? |
| Section 5: Swallowing |
| 23. Who screens for swallowing problems on the intensive care unit? |
| 24. How are swallowing problems determined on ICU? |
| 25. What clinical signs do you consider are evidence of swallowing problems in patients with a cervical spinal cord injury? |
| 26. What are the criteria for referring to Speech and Language Therapy? |
| 27. What swallowing assessment/s does the Speech & Language Therapist routinely use for patients on ICU? |
| 28. Do you consider it safe to allow a patient to eat and drink whilst they have the cuff inflated on their tracheostomy tube? |
| Section 6: Mouthcare |
| 29. Are you involved in the delivery of mouthcare?  If yes 🡪 31 |
| 30. Who is responsible for oral hygiene on your unit? |
| 31. How is the frequency of oral care determined? |
| 32. Do you advise on the following aspects of oral hygiene? |
| Section 7: Communication |
| 33. How do you support patients with a tracheostomy and cervical spinal cord injury who cannot speak in ICU? |
| 34. Is cuff deflation considered a communication option for ventilator dependent patients? |
| 35. Do you use in­line speaking valves with ventilator dependent patients? |
| Section 8: Comments |
| 36. Do you have any other comments you would like to add about the clinical management of cervical spinal cord injured patients? |
| 37. If you would like to be involved in a future aspect of the study to develop a screening tool, please add your email address: |
