# Supplementary material for: Oropharyngeal dysphagia management in cervical spinal cord injury patients: an exploratory survey of variations to care across specialised and non-specialised units
Source: Spinal Cord Ser Cases. 2019 Apr 15;5:31. doi: 10.1038/s41394-019-0175-y (PMC6474233; doi:10.1038/s41394-019-0175-y)
Supplement: Supplementary file 2 — Online survey questions [file 41394_2019_175_MOESM2_ESM.pdf]

RISCI
Respiratory Information for Spinal Cord Injury UK

Weaning guidelines for Spinal Cord Injured patients in Critical Care Units

Introduction

- It is an unfortunate fact that Spinal Cord Injury Centres have limited resources to accept ventilated patients. These guidelines are intended to aid the ventilator weaning process to enable faster transfer out of critical care areas.
- Spinal cord injured patients undergo physiological changes with time which tend to enable weaning in the majority.
- The weaning technique advocated by Spinal Cord Injury Centres is simple but needs to be followed rigorously to achieve ventilator independence efficiently. Weaning to complete ventilator independence can take up to several months.
- A few patients will remain ventilator dependant and there are processes by which verbal independence and in some, safe swallowing should be achieved.
- These guidelines are aimed primarily at adults.

Background pathophysiology

Respiratory dysfunction immediately following spinal cord injury is due to flaccid paralysis of respiratory muscles both inspiratory and expiratory. The degree of dysfunction is directly related to the level of cord injury.

Lumbar cord injuries will lose some expiratory abdominal activity.

Thoracic cord injuries will additionally lose intercostal activity and will frequently be complicated by rib fractures and pulmonary contusions. Haemothoraces may be present secondary to the thoracic spine fractures.

Low cervical cord injuries will have lost all intercostal activity.

High cervical injuries may also lose diaphragmatic and scalene activity. Ventilatory failure is rapid in these circumstances.

Autonomic disruption following on from cord injuries causes excessive bronchial secretions and a tendency to bronchoconstriction.

Some respiratory afferent information is lost; patients may not feel dyspnoeic or become tachypnoeic when failing.

Respiratory failure results from ineffective ventilation from compromised respiratory muscles acting on a flaccid rib cage aggravated by intrapulmonary compliance changes and an inability to spontaneously clear secretions.

It is occasionally possible using aggressive physiotherapy techniques and non invasive ventilation to support patients until pulmonary compliance improves to the point that unsupported ventilation is possible, but more commonly ventilatory failure occurs from minutes to days post injury requiring intubation and ventilation.

The physiological processes by which weaning becomes feasible include:

- Resolution of cord oedema. It is common for the neurological level to improve slightly with time which may allow use of previously paralysed respiratory muscles.

- Resolution of pulmonary pathology. **Pulmonary compliance needs to be as normal as possible for successful weaning.**

- Development of spasticity. Return of intercostal tone reduces chest wall compliance and improves ventilatory mechanics.

- Retraining of remaining functioning respiratory muscles.

Tracheostomy

Once intubated we recommend early tracheostomy as successful early extubation is rare.

Tracheostomy simplifies weaning, abolishes the need for sedation, improves communication and enables efficient secretion clearance.

There is no preference for percutaneous over surgical tracheostomy except with unstable cervical fractures where a surgical technique may cause less vertebral movement.

Tube changes for those patients requiring long term tracheostomies may be easier following surgical tracheostomy.

- 8 mm internal diameter tubes are optimal in adults.
- Removable inner cannulae are recommended in the early stages.
- Subglottic suction tubes may be of considerable benefit.
- There is no evidence of benefit for fenestrated tubes but there is evidence that they are associated with overgranulation

Pre requisites for weaning:

- Good pulmonary compliance: 50 ml/cm H_2O or greater
- $FiO_2 < 0.4$
- PEEP preferably around 5 cm H_2O
- Awake and cooperative. Minimal opiates. Preferably no delirium
- No active sepsis
- Some evidence of spontaneous respiratory activity.
    - **Ventilator triggering does not necessarily imply useful activity.**
    - Many patients will appear to pass spontaneous breathing trials early following injury, but rapidly develop respiratory fatigue requiring re-ventilation.
- Involved staff. Weaning proceeds more efficiently if a team of interested staff take control of the process.

Initial testing.

The premise for weaning is that some respiratory activity is present but weak, and a degree of respiratory muscle retraining is required.

The easiest and most reproducible measure of lung function for this is the vital capacity (VC). In the presence of low flows and low volumes a mechanical Wrights spirometer tends to perform better than electronic spirometers.

The vital capacity manoeuvre needs to be made by a cooperative patient completely free from ventilatory support. If still on relatively high PEEP a few breaths before the measurement is performed is advised.

A vital capacity as low as 150 mls is considered adequate to start weaning. A vital capacity approaching 1000ml predicts straightforward weaning.

With cord injuries at C4 and above, if there is doubt as to whether diaphragm activity is present, apnoea testing under sedation may be performed. This may show accessory muscle activity (Nasalis, sternomastoid) when the $PaCO_2$ rises above 6 Kpa without diaphragmatic activity if the cord injury involves the phrenic nerves. This does not necessarily imply permanent ventilator dependence but requires retesting at a later date.

Weaning principle

Based on the initial vital capacity measurement all ventilatory support is removed for a specified time and then re-instituted for a rest period. The common term for this is ventilator free breathing (VFB).

Suggested VFB times based on VC are:

1. If VC is less than 250 mls, start with 5 mintues VFB.
2. If VC is less than 500 mls, start with 15 minutes VFB.
3. If VC is greater than 750 mls, start with 30 minutes VFB

(Southport SCI unit)

- The on-ventilator rest period should be at least 1-2 hours. Trials of VFB can be repeated during day time hours, as appropriate to patient status.

- Weaning progression is achieved by increasing VFB time by specified amounts dependant on the previous day's results.

- It is important that the patient is not fatigued which can be estimated by re-measuring the VC at the end of the VFB period. If it is less that 70% of the pre weaning VC then either the rest period should be extended or the VFB time reduced.

For Example:

If a patient with a VC of 200 mls successfully achieves 3 episodes of 5 minutes VFB with 2 hour rest periods on day 1, with an end VFB VC of 180 mls, then increase the VFB time by 20% (to 6 mins) for day 2. If day 2 is satisfactory increase by 20% (8 mins) for day 3.

The initial aim is for VFB up to 18 hours during daytime, but for ventilation at night, as spinal cord injured patients can have significant REM sleep hypoventilation. To asses safe VFB overnight requires either $PaCO_2$ or $TcCO_2$ monitoring.

Adjuncts to weaning.

- Biochemistry and nutrition should be addressed. It is recommended that cervical cord injured patients and potential slow weaners have gastrostomies inserted.

- Regular salbutamol nebulisation may improve respiratory muscle function.

- VFB periods should be performed **supine, not sitting.** There is a drop of up to 20% in VC from supine to sitting, so VFB periods will be better tolerated supine. Secretion clearance should be performed prior to VFB periods. Tenacious sputum may be treated with oral/PEG carbocysteine or nebulised acetylcysteine.

- There is some evidence that during rest ventilation periods, high tidal volume ventilation whilst maintaining normocarbia accelerates weaning as it may reduce atelectasis.

Tracheostomy cuff deflation.

For all spinal cord injured patients the ability to communicate is paramount to rehabilitation and reintegration. Being in a critical care unit for considerable amounts of time without easy communication is at best frustrating and can contribute to psychological morbidity.

Cuff deflation can be achieved either on or off ventilation. Not only does this enable speech but also reduces microaspiration, restores laryngeal and pharyngeal reflexes leading to resumption of safe swallowing.

Off ventilator cuff deflation during VFB for fast weaners should be considered. If a subglottic suction tracheostomy is in place then this should be aspirated, otherwise a tracheal suction catheter placed to catch pooled saliva as it passes the deflating cuff. When deflated a speaking valve should be used, (if there is sufficient insufflation leak – if not consider downsizing) preferably a Passy Muir as they have favourable mechanics for spontaneously breathing low volume patients.

The use of a speaking valve introduces an element of PEEP which may improve respiratory mechanics and reduce the development of atelectasis.

On ventilator cuff deflation should be considered for slow weaners. Ventilator settings should be adjusted to allow for the resultant leak, either increases in IPAP or inspiratory time. Many ventilators will alarm continuously with this degree of leak so a change to simpler, domicillary type device can be considered – contact your Spinal Cord Injury Centre to ask what machine they use.

Many patients develop increased leaks when asleep, requiring partial or full cuff inflation in order to achieve adequate ventilation.

Optimal practice is to change cuffed for uncuffed tubes wherever possible when cuffs can be deflated for 24 hours.

Swallowing.

Attempts at swallowing with an inflated tracheostomy cuff are never safe. It is advisable to wait until cuff deflation is achieved and enlist the advice of a speech and language therapist.

Post weaning maintenance

Patients who have successfully weaned or who are ventilator free during the day are still at risk of respiratory decompensation.

Functional residual capacity and inspiratory muscle strength continue at a reduced level.

Intermittent IPPB or manual hyperinflation are of benefit in reducing atelectasis.

Further information

All UK Spinal Cord Injury Centres have someone with an interest in respiratory management. Contacts can be found at www.risci.org.uk

RISCI is a multi-disciplinary group concerned with standards of care provided for spinal cord injured patients requiring respiratory support before and during admission to a Spinal Cord Injury Centre, and after discharge.

Version control

**Rf/KH JR TT amended**	**9th May 2012**	
Formatted for NSCISB	10th May 2012	
